# Persistent Amyloidosis following Suppression of Aβ Production in a Transgenic Model of Alzheimer Disease

**DOI:** 10.1371/journal.pmed.0020355

**Published:** 2005-11-15

**Authors:** Joanna L Jankowsky, Hilda H Slunt, Victoria Gonzales, Alena V Savonenko, Jason C Wen, Nancy A Jenkins, Neal G Copeland, Linda H Younkin, Henry A Lester, Steven G Younkin, David R Borchelt

**Affiliations:** **1**Department of Pathology, The Johns Hopkins University School of Medicine, Baltimore, Maryland, United States of America,; **2**Division of Biology, California Institute of Technology, Pasadena, California, United States of America,; **3**Department of Cell Biology, The Johns Hopkins University School of Medicine, Baltimore, Maryland, United States of America,; **4**Mouse Cancer Genetics Program, National Cancer Institute Frederick Cancer Research and Development Center, Frederick, Maryland, United States of America,; **5**Mayo Clinic Jacksonville, Jacksonville, Florida, United States of America,; **6**Department of Neuroscience, The Johns Hopkins University School of Medicine, Baltimore, Maryland, United States of America; Zürich UniversitySwitzerland

## Abstract

**Background:**

The proteases (secretases) that cleave amyloid-β (Aβ) peptide from the amyloid precursor protein (APP) have been the focus of considerable investigation in the development of treatments for Alzheimer disease. The prediction has been that reducing Aβ production in the brain, even after the onset of clinical symptoms and the development of associated pathology, will facilitate the repair of damaged tissue and removal of amyloid lesions. However, no long-term studies using animal models of amyloid pathology have yet been performed to test this hypothesis.

**Methods and Findings:**

We have generated a transgenic mouse model that genetically mimics the arrest of Aβ production expected from treatment with secretase inhibitors. These mice overexpress mutant APP from a vector that can be regulated by doxycycline. Under normal conditions, high-level expression of APP quickly induces fulminant amyloid pathology. We show that doxycycline administration inhibits transgenic APP expression by greater than 95% and reduces Aβ production to levels found in nontransgenic mice. Suppression of transgenic Aβ synthesis in this model abruptly halts the progression of amyloid pathology. However, formation and disaggregation of amyloid deposits appear to be in disequilibrium as the plaques require far longer to disperse than to assemble. Mice in which APP synthesis was suppressed for as long as 6 mo after the formation of Aβ deposits retain a considerable amyloid load, with little sign of active clearance.

**Conclusion:**

This study demonstrates that amyloid lesions in transgenic mice are highly stable structures in vivo that are slow to disaggregate. Our findings suggest that arresting Aβ production in patients with Alzheimer disease should halt the progression of pathology, but that early treatment may be imperative, as it appears that amyloid deposits, once formed, will require additional intervention to clear.

## Abstract 2

In a transgenic mouse that overexpressed amyloid beta, turning off production via a tetracycline sensitive switch did not decrease the number of amyloid plaques present

## Introduction

Over a decade ago the amyloid cascade hypothesis predicted that increased levels of amyloid-β (Aβ) peptide lead to secondary pathologies that ultimately culminate in the onset of Alzheimer disease (AD) [1]. Early support for this hypothesis came from genetic studies linking early-onset AD to mutations in the amyloid precursor protein (APP), from which Aβ is derived, and presenilins 1 and 2, which are interchangeable components of a endoprotease complex that releases Aβ from APP (for review see [2,3]). If, as predicted, overproduction of Aβ initiates the cascade of events leading to disease, then therapeutic strategies that lower Aβ levels should either arrest or reverse the progression from peptide to dementia.

Early evidence from clinical trials of antibody-mediated clearance, one of the first Aβ-lowering approaches tested in humans, suggested that treatments designed to reduce amyloid burden may indeed be beneficial. Although the trials were halted because of adverse effects in a subset of volunteers [4,5], further analysis of several patients found evidence that amyloid pathology, and to a lesser degree cognitive function, was improved in proportion to the patient's titer of Aβ-specific antibody [6,7]. While this approach is promising, constant exposure to antibodies that recognize an epitope highly enriched in the brain may have unexpected side effects that will limit its long-term use.

An alternative approach that is being actively pursued for future treatment of AD seeks to lower Aβ levels by limiting its production from the precursor protein APP. Peptide Aβ is released from APP by the action of two enzymes, the β-APP cleaving enzyme 1 (BACE1) and γ-secretase, which cleave the holoprotein at the N- and C-termini of Aβ, respectively. Several inhibitors of γ-secretase have already been produced [8,9], and small molecule inhibitors of β-APP cleaving enzyme 1 are currently being developed [10,11]. The long-term effectiveness of this approach in either humans or model systems, however, has not been reported. Although loss of β-APP cleaving enzyme 1 function can prevent the development of plaques in transgenic mouse models for AD (F. Laird, H. Cai, P. C. Wong, personal communication), it is not known whether the brain can clear pre-existing amyloid deposits once production of Aβ has been suppressed.

Clearly, the amyloid-lowering approach should be rigorously examined in animal models before these reagents are tested in patients. However, the chemical secretase inhibitors most likely to reach human trials are still in development. Therefore, we developed a mouse model of Alzheimer-type amyloid that expresses a controllable APP transgene. This system, commonly known as the tet-off system, can be regulated by analogs of tetracycline administered in food or water [12,13]. The strong expression levels produced with the tet-off vectors, combined with the ability to reduce this expression by several orders of magnitude with tetracycline [14], allowed for a stringent test of how a highly effective pharmaceutical inhibitor of Aβ production would impact the progression of amyloid pathology and whether reversal of these lesions might be possible following such treatment.

## Methods

### Transgene Construction

We created a tetracycline-responsive chimeric mouse/human APP695Swedish/Indiana (swe/ind) vector by replacing the promoter region of the moPrP.XhoI vector (also known as pPrPpE1/E2,3sal [15]) with the tetracycline-responsive promoter of pTetSplice (Life Technologies, Rockville, Maryland, United States), and then ligating mouse APP with a humanized Aβ domain (mo/huAPP695) cDNA into the new vector. We began by cloning the tetracycline-responsive promoter (bp 6–481) from pTetSplice by PCR using primers that added external BamHI and NotI sites to the 5′ end and a BamHI site to the 3′ end, while destroying XhoI and BamHI sites within the promoter (forward: GCC GGA TCC GCG GCC GCC GTC GAG TTT ACC ACT CCC TAT C; reverse: GCC GGA TCC ACT CTA GAA GAT CCC CGG GTA CCG). We then isolated the moPrP.XhoI intron by amplification with primers that added an external BamHI site to the 5′ end of exon 1 and ran through the Asp718 site of exon 2 (forward: GCC GGA TCC GAT CAG CAG ACC GAT TCT GG; reverse: GCC GGT ACC ACT AGG AAG GCA GAA TGC). This 2-kb intron fragment was cloned into a TA cloning vector (Invitrogen, Carlsbad, California, United States), then excised by Asp718 digestion and ligated to the 6.8-kb Asp718 fragment of moPrP.XhoI containing exon 2, exon 3, the 3′ UTR, and pBluescript to generate an intermediate vector with all three exons and a central intron but no promoter. This vector was then opened at the BamHI site introduced by the intron cloning primer, and ligated to the 0.5-kb BamHI-cut tetracycline promoter fragment. This ligation generated a 9.3-kb vector encoding the tetracycline promoter from pTetSplice with two exons, one intron, and the original 3′ UTR of the moPrP.XhoI vector, all carried in the pBluescript cloning vector.

We incorporated the Swedish (KM570/571NL) and Indiana (V617F) mutations into the mo/huAPP695 cDNA (in BS-KS) by PCR using a four-primer strategy: first, two partially overlapping products were generated in separate reactions using primers that encode the desired mutations (Swedish forward: GGA GAT CTC TGA AGT GAA TCT GGA TGC AGA ATT CCG/Indiana reverse: GGG TGA TGA AAA TCA CGG TTG C; Indiana forward: CAA CCG TGA TTT TCA TCA CCC TGG/M13 reverse). The two PCR products were ligated, digested with BglII and ApaI and cloned back into the original mo/huAPP695-BS-KS vector. Finally, the new APP695swe/ind was subcloned into the XhoI site of the moPrP-tetP vector from above to complete the construct.

### Pronuclear Injection, Screening of Founders, and Maintenance of the Lines

The moPrP-tetP-mo/huAPP695swe/ind vector was linearized and the pBluescript domain excised by digestion with NotI. The purified vector was injected into the pronucleus of fertilized eggs from C57BL/6J × C3HeJ F1 matings. Founder animals were screened for the presence of the transgene by three-way PCR using the S36 and PrP-S/PrP-AS primers described below. Transgene-positive founders were bred to animals expressing the tetracycline transactivator (tTA) under control of the calcium-calmodulin kinase IIα (CaMKIIα) promoter obtained from Jackson Laboratory [16] (Bar Harbor, Maine, United States; stock # 3010; B6;CBA-TgN[Camk2a-tTA]1Mmay). The colony was thereafter maintained by crossing single transgenic tTA and APP offspring for each of the four APP lines. All mice were provided fresh food and water ad libitum. Animal protocols were approved by both the Johns Hopkins University and the California Institute of Technology Institutional Animal Care and Use Committees.

### Doxycycline Administration

Doxycycline (dox) was administered through commercially available dox-containing chow (BioServ, Frenchtown, New Jersey, United States). The chow contained 200 mg/kg of antibiotic; based on estimated consumption of 5 g per mouse per day, the expected dose to each animal was 1 mg dox per day. The average 25-g animal therefore received 40 μg of dox per gram body weight per day. Chow was changed 1–2 times per week to prevent breakdown of the antibiotic.

### Genotyping

Offspring were genotyped for the presence of each transgene by PCR amplification of genomic DNA extracted from a 5-mm tail biopsy. Tails were heated to 95 °C for 45 min in 250 μl of 50 mM NaOH, vortexed, then neutralized with an equal volume of 0.5 M Tris-HCl (pH 5.5). Debris was sedimented by centrifugation, and 3 μl of supernatant was used for amplification.

Genotyping for APP and tTA transgenes was performed in the same PCR reaction, using five separate primers. APP was amplified using forward primer S36 located in the 3′ end of the APP cDNA (CCG AGA TCT CTG AAG TGA AGA TGG ATG) and reverse primer PrP-AS-J located in the 3′ UTR of the vector (CCA AGC CTA GAC CAC GAG AAT GC). The tTA transgene was detected using a primer set that amplified across its two subdomains with tet forward located within the Tn10 tetracycline repressor (CGC TGT GGG GCA TTT TAC TTT AG) and tet reverse within the HSV1 VP16 (CAT GTC CAG ATC GAA ATC GTC). All reactions, whether transgene-positive or not, amplified a segment of the endogenous prion protein gene as a control for DNA quality using a forward primer, PrP-S-J, specific to the mouse PrP open reading frame (GGG ACT ATG TGG ACT GAT GTC GG) and a reverse primer, PrP-AS-J, shared by the 3′ UTR of the endogenous PrP gene and the transgene vector. Amplification reactions were run for 37 cycles at 94 °C for 30 s, 64 °C for 1 min, and 72 °C for 1 min. All samples, transgenic and wild-type, gave a 750-bp product from the endogenous PrP gene. The APP transgene yielded an additional band at 400 bp; the tTA product fell in between at 480 bp.

### Immunoblotting/Quantitation

Frozen cortical or whole forebrain tissue was homogenized by sonication in five volumes of phosphate-buffered saline (PBS) with 5 mM EDTA and protease inhibitors (Mammalian cell cocktail, Sigma, St. Louis, Missouri, United States), using a probe sonicator set to 50% output (TEKMAR, Cincinnati, Ohio, United States). After dilution with an equal volume of PBS/EDTA/protease inhibitor, the samples were centrifuged briefly and the supernatant used for analysis. Fifty micrograms (6E10 and CT15) or 5 μg (22C11) of brain homogenate was loaded per lane onto 7.5%, 10%–20%, or 4%–20% Tris-HCl PAGE gels (Bio-Rad Laboratories, Hercules, California, United States) and electrophoresed for several hours in 1× Tris-glycine–sodium dodecyl sulfate (1×TG-SDS) buffer (6E10 and 22C11; Amresco, Solon, Ohio, United States) or 1× Tris-tricine-SDS buffer (CT15; Invitrogen, Carlsbad, California, United States). Proteins were transferred overnight to 0.45-μm Optitran nitrocellulose (Schleicher and Schuell, Keene, New Hampshire, United States) in 1× TG buffer (Amresco). Blots were blocked in PBS containing 5% nonfat dry milk powder, and incubated for 3 h at room temperature in blocking solution with one of the following antibodies: mouse monoclonal 22C11 (kind gift of Konrad Beyreuther and Andreas Weidemann; [17]) diluted 1:1,000, mouse monoclonal 6E10 (Signet Laboratories, Dedham, Massachusetts, United States) diluted 1:2,500, rabbit polyclonal anti-superoxide dismutase 1 (m/hSOD1) [18] diluted 1:2,500 to 1:4,000, or rabbit polyclonal CT15 (kind gift of Ed Koo; [19]) diluted 1:1,000. Subsequently, the blots were washed with PBS containing 0.1% Tween-20, and then incubated with either goat anti-mouse– or goat anti-rabbit–HRP conjugated secondary antibody diluted 1:1,000 in blocking solution. After several additional rinses in PBS with 0.1% Tween-20, blots were developed with enhanced chemiluminescence reagent and imaged with the Bio-Rad Molecular Imager FX system.

Staining intensity within each lane was quantified using the Quantity One image analysis software (Molecular Imager FX, Bio-Rad Laboratories). Background was calculated from across the image and subtracted from the entire file. The signal intensity for each band (corrected signal intensity × pixel number) was then calculated using the Volume report tool.

### Slot Blot mRNA Analysis

Five micrograms per sample of total RNA extracted from fresh-frozen brain, liver, kidney, heart, lung, spleen, and skeletal muscle was vacuum-filtered through 0.45-μm Optitran nitrocellulose. After several washes through the manifold with 10× SSC, blots were UV-cross-linked and probed with a radiolabeled ∼350-bp BglII–XhoI cDNA fragment of mo/huAPP695 cDNA. After hybridizing overnight at 65 °C in 1% BSA/1 mM EDTA/0.5 M sodium phosphate buffer (pH 7.2)/7% SDS [20], the blots were washed twice at 65 °C for 30 min each in 0.1% BSA/1 mM EDTA/40 mM sodium phosphate buffer (pH 7.2)/5% SDS before two final 30-min washes at 65 °C with 1 mM EDTA/40 mM sodium phosphate buffer (pH 7.2)/1% SDS. Blots were wrapped wet and exposed to phosphorscreens overnight at room temperature.

### Amyloid Histology

Mice were euthanized by ether inhalation and brains removed for immersion fixation in 4% paraformaldehyde/1× PBS. After 48 h in fixative at 4 °C, brains were transferred to PBS, dehydrated in alcohols, treated with cedarwood oil and methylsalicylate, and embedded in paraffin for sectioning.

#### Hirano silver stain

Silver impregnation histology was performed on 10-μm paraffin-embedded sections by Hirano's modification of the Bielschowsky method [21]. Briefly, sections were deparaffinized through xylene and alcohols into tap water before being placed into fresh 20% silver nitrate solution for 20 min. After being washed thoroughly with distilled water, slides were immersed in 20% silver nitrate solution titrated with fresh ammonium hydroxide. After 20 min, slides were washed with ammonia water before being individually developed with 100 μl of developer (20 ml of 37% formaldehyde, 100 ml of distilled water, 50 μl of concentrated nitric acid, and 0.5 g of citric acid) added to 50 ml of titrated silver nitrate solution. Slides were then rinsed in tap water, fixed in 5% sodium thiosulfate, and dehydrated through alcohols and xylene.

#### Thioflavin-S staining

Following deparaffinization of sections through xylene and alcohols, amyloid impregnation with thioflavin-S was performed according to the Guntern modification of the standard protocol. Slides holding 10-μm paraffin sections were washed twice in distilled water, then immersed for 5 min in a 0.25% potassium permanganate solution, followed by 5 min in a 1% potassium metabisulfate/1% oxalic acid solution. After this preparation, slides were placed into a filtered aqueous 0.02% thioflavin-S solution (Chroma-Gesellschaft Schmid, Kongen, Germany) for 8 min. Excess stain was removed by two brief rinses in 80% ethanol, then two in distilled water, after which slides were finished in aqueous mounting medium for florescence photomicrography.

#### Ubiquitin, glial fibrillary acidic protein, and Aβ immunohistochemistry

Prior to immunostaining, slides were deparaffinized by oven heating followed by immersion in xylene. After rehydration through graded alcohols into tap water, endogenous peroxidase activity was quenched by incubation with 3% hydrogen peroxide in methanol. Slides were microwaved for 5–7 min in water, cooled for 5 min, then washed in TBS. Nonspecific staining was blocked for 1 h with 3% normal goat serum and 0.1% Triton-X 100 in TBS. Slides were then placed into primary antibody (rabbit anti-Aβ peptide polyclonal antibody, Zymed Laboratories, South San Francisco, California, United States; rabbit anti-ubiquitin and rabbit anti–glial fibrillary acidic protein (GFAP) polyclonal antibodies, Dako, Carpinteria, California, United States) diluted 1:500 in TBS with 2% normal goat serum and incubated overnight at room temperature. After being washed of excess primary antibody with several changes of TBS, slides were incubated with either the Vectastain Elite anti-rabbit secondary system (anti-Aβ; Vector Laboratories, Burlingame, California, United States) or peroxidase/anti-peroxidase reagents (anti-ubitquitin and anti-GFAP; Sternberger Monoclonals, Lutherville, Maryland, United States) according to the manufacturers' directions. Antibody binding was visualized with diaminobenzidene, and sections were counterstained with hematoxylin.

### Filter Trap Assay

An aliquot of each cortical homogenate used for Western blotting above was partially solubilized by the addition of SDS to a final concentration of 1%. Serial 1:2 dilutions were made with 1× PBS/1% SDS, and 100 μl of each dilution was then vacuum-filtered through a pre-wet 0.22-μm cellulose acetate membrane (Schleicher and Schuell) [22]. Each well was washed several times with PBS, after which blots were incubated overnight with polyclonal anti-Aβ antibody (Zymed Laboratories) diluted 1:600 in a blocking solution of 1× TBS/5% nonfat dry milk powder. After washing the blots three times for 10 min each in 1× TBS/0.1% Tween-20, the membrane was incubated for 1 h with HRP-conjugated protein A (Sigma) diluted 1:5,000 in blocking solution. The membranes were again washed three times with 1× TBS/0.1% Tween-20, before antibody binding was detected with enhanced chemiluminescence (PerkinElmer, Boston, Massachusetts, United States). Digital images of each blot were captured with a Molecular Imager FX gel documentation system, and the intensity of Aβ staining was quantified using Quantity One image analysis software.

### Aβ ELISA

An aliquot of cortical homogenate generated for Western analysis described above was subjected to a three-step sequential extraction using PBS, 2% SDS, and 70% formic acid (FA). At each step, the sample was sonicated in appropriate buffer and centrifuged at 100,000*g* for 30 min (1- to 1.5-mo samples) or 60 min (6- to 12-mo samples) at 4 °C as previously described [23–25]. The supernatant was removed for analysis, and the pellet was sonicated in the next solution in sequence. The 2% SDS extracts were diluted in EC buffer, and the FA extracts neutralized with 1M Tris-phosphate buffer (pH 11) then diluted with EC buffer prior to testing. Human Aβ was measured in each fraction using BAN50 for capture (epitope Aβ1–16) and BA27 and BC05 for detection (Aβ40 and Aβ42, respectively) (Takeda Chemical Industries, Osaka, Japan). Total Aβ (mouse + human; 1- to 1.5-mo samples only) was measured in each fraction using BNT77 for capture (epitope Aβ11–28) and BA27 and BC05 for detection. All values were calculated as picomoles per gram based on the initial weight of cortical tissue.

### Activity Monitoring

Daily basal activity was studied in 28 CaMKIIα-tTA × tet-APPswe/ind line 107 mice between 4 and 5 mo of age. Animals were separated into individual cages immediately before the start of each experiment (*n =* 3–6 per genotype untreated, 2–5 per genotype dox-reared). The cages were placed inside activity-monitoring frames designed to count every time the animal passed through one of three photobeams spanning the width of the cage (San Diego Instruments, San Diego, California, United States). Experiments were started midway through the light phase of the day, and data were collected in 1-h bins for the following 48 h. Testing rooms were maintained on the same 13:11 h day:night cycle as the main animal housing areas and were closed to entry during the experiment.

### Statistical Analyses

Statistical analyses of protein expression, ELISA data, and filter trap assays were performed by ANOVA with Tukey's honest significant difference post-hoc test applied to significant main effects or interactions (Statistica 6.0, StatSoft, Tulsa, Oklahoma, United States). In cases of positively skewed data distribution, log10(*x* + 0.5) transformation was applied to the raw data before submitting them to ANOVA.

## Results

**Figure 1:**
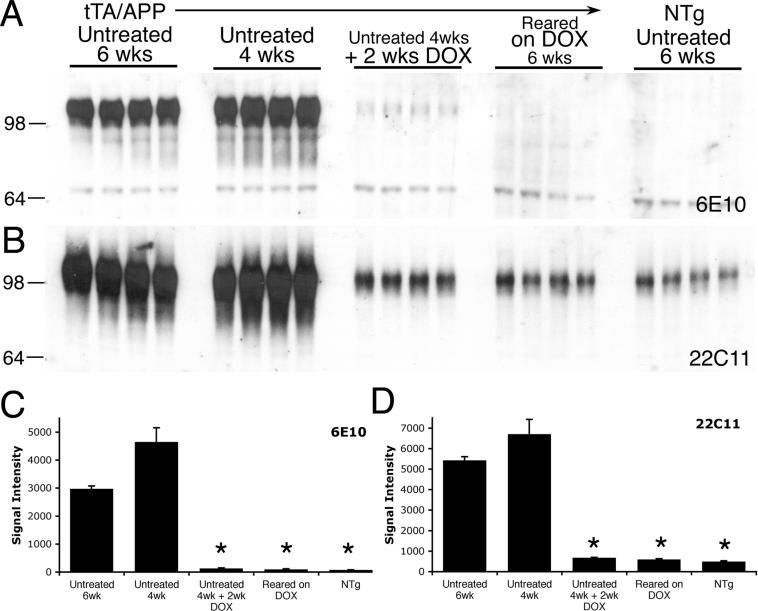
Control of Transgenic APP Expression by Dox (A) Western blotting for transgenic APP using the human-specific 6E10 antibody shows expression of full-length protein in forebrain tissue from young predeposit double transgenic animals (line 107) and its suppression following dox treatment. Untreated animals show high levels of transgene expression; protein levels drop dramatically in animals acutely treated with dox for 2 wk. A faint, but detectable band of full-length protein remains in acutely treated animals that can be eliminated in mice born and raised on dox. (B) Immunoblotting with the N-terminal antibody 22C11 to detect both transgenic and endogenous protein shows that dox treatment reduces APP/APLP to levels found in nontransgenic mice. (C) Measurement of signal intensity from the Western blot in (A) shows transgenic APP levels are decreased more than 95% by dox in both acutely and chronically treated animals (97.2% for 4 wk + 2 wk dox, 98.0% for reared on dox versus 4 wk untreated; ANOVA effect of treatment group *F*_4,15_ = 85.55, *p <* 0.001). APP levels in 4 wk + 2 wk dox, reared on dox, and nontransgenic (NTg) were not significantly different (*p >* 0.9, Tukey post-hoc test). (D) Measurement of signal intensity from the Western blot in (B) shows total APP/APLP levels in dox-treated tTA/APP mice are significantly lower than in 4-wk-old untreated mice (ANOVA effect of treatment group *F*_4,15_ = 84.41, *p <* 0.001) and indistinguishable from those of nontransgenic animals (*p >* 0.9, Tukey post-hoc test). *, *p <* 0.001 versus untreated 4-wk-old mice, Tukey post-hoc test applied to significant effect of group ANOVA.

We used the tet-off transgene system to express a double mutant version of chimeric mo/huAPP695 (swe/ind KM570, 571NL, and V617F) from a tetracycline-responsive promoter [12,13]. Transgenic APP expression was activated by crossing the APPswe/ind mice to animals producing tTA under control of the CaMKIIα promoter [16]. After initial screening of founders, we identified four lines of tet-APPswe/ind mice that produced very high levels of transgene product in offspring coexpressing tTA (Figures 1 and Figure S1). Compared to a standard APP transgenic line used for previous amyloid studies by our laboratory (line C3–3; [15,26,27]), we estimated that the four controllable lines produce transgenic APP protein at 10- to 30-fold over endogenous levels (Figure S1). This estimate was confirmed by direct comparison of APP levels in nontransgenic and tet-off APP mice using an antibody that recognizes both endogenous APP (and amyloid precursor-like protein 2) and the transgenic protein (monoclonal antibody 22C11; Figure 1D).

Importantly, all four new lines of tet-off APP mice showed nearly complete suppression of the transgene following dox treatment (Figures 1 and Figure S1). We focused on one of the four lines, line 107, to examine in more detail the time dependence and extent of transgene suppression following either acute or chronic treatment with dox. Two dox-treated groups were compared to two untreated groups: one group of mice was born and raised on dox, a second group was treated with dox for 2 wk starting at 1 mo of age (4 wk + 2 wk dox); two untreated groups kept on normal chow were harvested at either 4 or 6 wk of age. Animals born and raised on dox harbored no transgenic APP (Figure 1A). Following as little as 2 wk of dox treatment, transgenic APP expression was reduced by more than 95% compared to pre-dox levels. The residual expression remaining in acutely treated mice represents less than 4% of the transgenic protein produced in the absence of dox (Figure 1C), and likely results from slight leakage at the level of transcription (data not shown). Importantly, the total amount of APP (endogenous plus transgenic) and related APLPs in both acute and chronically treated animals was statistically indistinguishable from that in nontransgenic mice (Figure 1D; statistical analyses for experiments throughout the study are presented in the accompanying figure legends).

**Figure 2:**
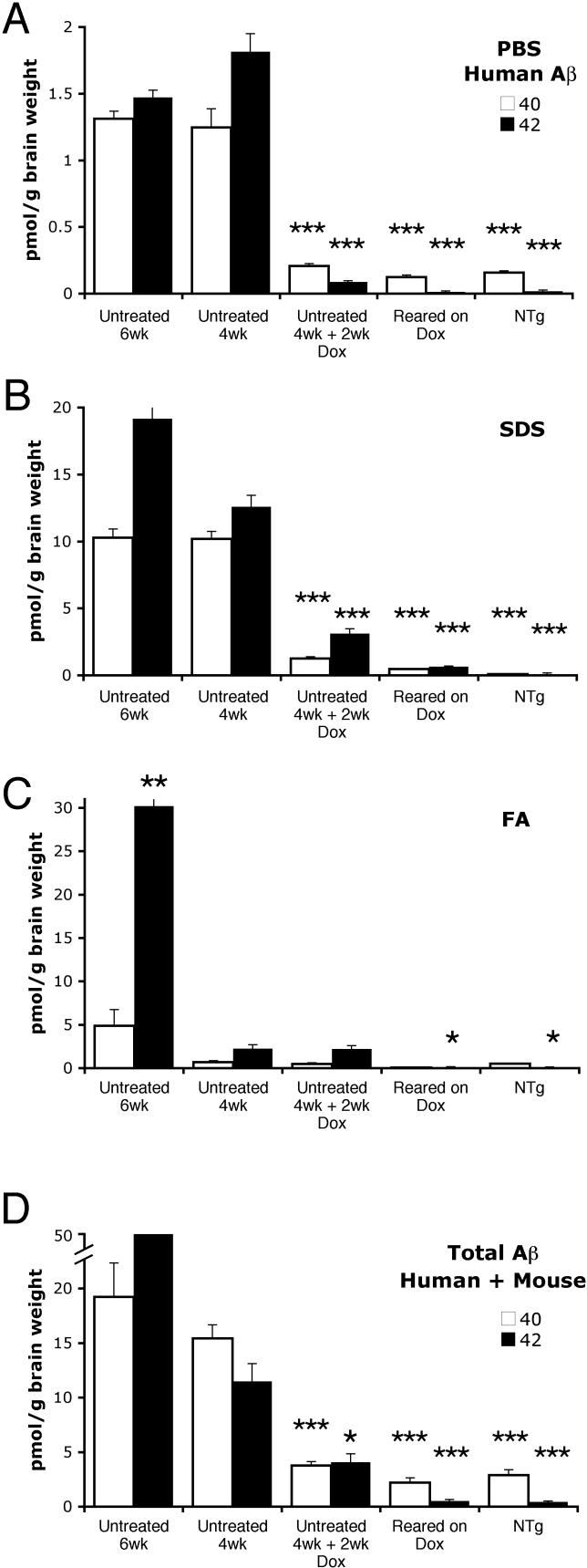
Aβ Levels Are Dramatically Reduced by Transgene Suppression Cortical homogenates from young, predeposit tTA/APP mice used for Western blot in Figure 1 (line 107) were fractionated by sequential multi-step extraction with PBS, 2% SDS, and 70% FA followed by human-specific Aβ ELISA to measure transgene-derived peptide in each fraction. Aβ40 is shown in white, Aβ42 in black. (A) PBS-soluble Aβ levels are substantially reduced by both acute and chronic dox treatment (ANOVA, effect of treatment group *F*_4,24_ = 137.10 and 386.01, *p <* 0.001, for Aβ40 and Aβ42, respectively). Aβ levels in treated animals are indistinguishable from nontransgenic (NTg) animals (*p >* 0.3, Tukey post-hoc test). (B) In the young animals tested here prior to the formation of visible amyloid deposits, most Aβ is extracted into the SDS fraction (84% and 76% of all transgene-derived Aβ40 and Aβ42, respectively). As in the PBS-soluble fraction, Aβ levels in the SDS fraction are significantly lowered by dox treatment compared to untreated animals (ANOVA effect of group *F*_4,24_ = 197.57 and 163.48, *p <* 0.001, for Aβ40 and Aβ42, respectively). Acutely treated animals retained a small (although significant) amount of residual peptide (*p <* 0.001 compared to nontransgeinc, Tukey post-hoc test), whereas Aβ levels in mice born and raised on dox were reduced to levels indistinguishable from nontransgenic (*p >* 0.8, Tukey post-hoc test). (C) The FA-soluble fraction already contains a small but significant pool of aggregated Aβ42 in untreated animals by 4 wk of age (*p <* 0.05 versus nontransgenic; Tukey post-hoc test applied to significant effect of group ANOVA *F*_4,24_ = 17.11, *p <* 0.001). By 6 wk of age, the amount of Aβ in the FA fraction is increased significantly preceding the appearance of visible deposits 2 wk later. The FA pool is the only peptide fraction not lowered by acute dox treatment (4 wk untreated = 4 wk + 2 wk dox, *p >* 0.9, Tukey post-hoc test), consistent with poor turnover of aggregated Aβ species. (D) Measurements of total Aβ, including both endogenous and transgene-derived peptides, show that animals born and raised on dox harbor Aβ levels identical to nontransgenic animals (*p >* 0.9, Tukey post-hoc test, effect of group ANOVA *F*_4,24_ = 39.13 and 35.29, *p <* 0.001, for Aβ40 and Aβ42, respectively). Whereas chronic transgene suppression fully prevents synthesis of both peptides, acute dox treatment fully suppresses Aβ40 levels (*p >* 0.8, Tukey post-hoc test), but leaves a small amount of nonsuppressed Aβ42. The residual Aβ42 observed in acutely treated young animals derives from uncleared aggregates extracted in the SDS and FA fractions. *, *p <* 0.05; **, *p <* 0.005; ***, *p <* 0.001 versus 4-wk-old untreated mice, Tukey post-hoc applied to significant effect of group ANOVA.

To ensure that Aβ production was suppressed in concert with the dox-mediated inhibition of its precursor APPswe/ind, we measured Aβ40 and Aβ42 levels by ELISA in forebrain homogenates from young tet-off animals. At 1 mo of age, the mice lacked visible amyloid aggregates that might act as an intractable reservoir of peptide remaining in the brain after the transgene had been suppressed. To further ensure we could detect any such insoluble aggregates that might bias our measure of changes in peptide synthesis, we performed a sequential three-step extraction with PBS, 2% SDS, and 70% FA that would separate peptides by solubility. We compared the levels of human transgene-derived Aβ40 and Aβ42 in untreated mice at 4 and 6 wk of age to animals that had either been born and raised on dox or that had been left untreated for 4 wk and then placed on dox chow for 2 wk prior to harvest (the same groups described above for immunoblot analysis of APPswe/ind levels, line 107). Consistent with the reduction in full-length APPswe/ind synthesis shown by immunoblot (see Figure 1), we found that transgene-derived Aβ levels were completely suppressed in animals born and raised on dox, and were sharply reduced following acute (2 wk) antibiotic treatment. Compared to the levels in untreated 4-wk-old mice, PBS-soluble Aβ42 dropped by 95.2% following 2 wk of dox treatment and by 99.2% with chronic treatment (Figure 2A). Similarly, SDS-soluble Aβ42 decreased by 75.2% and 94.8% following 2-wk or lifelong dox treatment (Figure 2B). Only the FA fraction revealed a small dox-resistant pool of peptide in acutely treated animals that we believe represents stable predeposit aggregates that have already accumulated by 4 wk of age when treatment was begun (Figure 2C). Indeed, animals that were born and raised on dox did not harbor this reservoir of treatment-resistant peptide, with 96.3% less Aβ42 than untreated 4-wk-old mice. Measurement of total Aβ in chronically treated mice, including endogenous and transgene-derived peptide, demonstrated that Aβ levels in tet-off APP mice were reduced to the level of endogenous peptide found in nontransgenic animals (Figure 2D). Taken together with the immunoblotting data for full-length APPswe/ind, the ELISA measurements indicate that dox-mediated suppression of transgenic APPswe/ind synthesis leads to parallel reduction of Aβ levels.

The ELISA data also confirmed that incorporation of the Swedish and Indiana mutations led to high levels of Aβ42, which we predicted would induce rapid plaque formation in untreated animals. Histological characterization of double transgenic (CaMKIIα-tTA × tet-APPswe/ind) mice revealed early-onset amyloid formation in all four new lines. Amyloid plaques were seen in mice as young as 8 wk of age (data not shown). Plaques were limited to the forebrain, including the cortex, hippocampus, olfactory bulb, and striatum, where the CaMKIIα promoter is known to be most active [16,28] (Figure S2). By 6 mo of age, amyloid burden became severe, covering large areas of the cortex and hippocampus (Figure S3). No lesions were seen in the cerebellum or brain stem even at late ages, consistent with CaMKIIα-controlled transgene expression. Unlike what is thought to occur in the human disease, the first visible plaques in the tet-off APP mice are fibrillar-cored deposits. We have noted the same early appearance of cored deposits in other lines of APP transgenic mice that harbor the Swedish mutation [27]. Diffuse plaques were apparent in 6-mo-old tTA/APP mice, and became relatively abundant by 9 mo of age. At older ages (9–12 mo) amyloid deposits were visible in the thalamus, which has also been observed in mice expressing mutant APP via the Thy-1 promoter. The presence of amyloid pathology in this region has been attributed to axonal transport of APP/Aβ to the terminals of cortical neurons in the thalamus [29]. Most importantly, only double transgenic mice, expressing both the tTA and APP transgenes, developed amyloid lesions. Single transgenic mice up to 15 mo of age showed no sign of pathology (Figure S3). Similarly, amyloid pathology can be completely prevented in double transgenic animals born and raised on dox. Animals from our highest expressing line (line 885) maintained on dox for up to 1 y harbored no amyloid pathology (data not shown), indicating that residual leakage of transgene expression in the presence of dox does not provide sufficient Aβ peptide to induce amyloid formation even over long periods.

**Figure 3:**
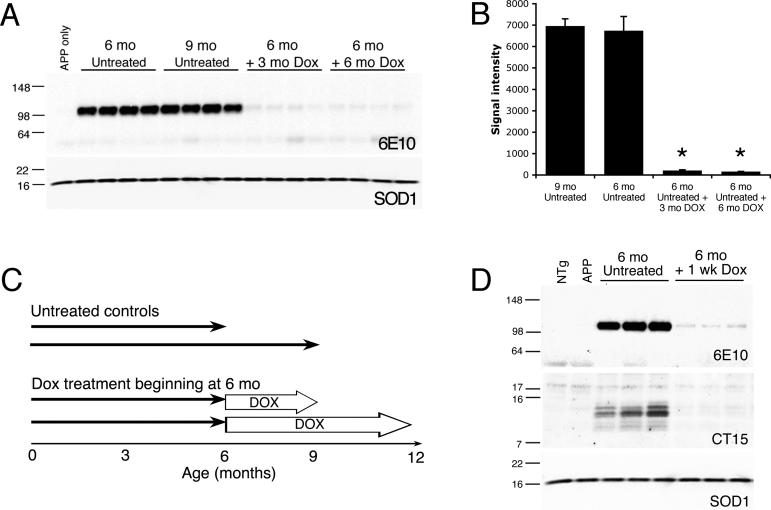
Robust Transgene Suppression in Older Mice with Preexisting Amyloid Pathology (A) Cortical homogenates from 6- to 12-mo-old animals used for pathology studies described below (line 107) were immunoblotted with human-specific antibody 6E10 to examine transgene suppression following 3 or 6 mo of dox treatment. The blot was co-immunostained for endogenous superoxide dismutase 1 (SOD1) as a control for loading. (B) Quantitation of signal intensity from the Western blot shown in (A). Transgenic APP levels are significantly suppressed following 3 or 6 mo of dox treatment (96.9% and 97.6%, respectively). *, *p <* 0.001 compared to 6-mo-old untreated animals, Tukey post-hoc test applied to significant effect of group ANOVA *F*_3,12_ = 107.22, *p <* 0.001. These data demonstrate that strong transgene suppression is attained both before and after the onset of amyloid pathology (see Figure 1 for predeposit experiments). (C) Experimental design. To examine the effects of chronic Aβ suppression on amyloid pathology after the onset of deposition, we compared untreated controls harvested at 6 and 9 mo of age to animals placed on dox at 6 mo of age and harvested after 3 or 6 mo of treatment. (D) Dox treatment leads to rapid transgene suppression even in 6-mo-old tTA/APP mice. Immunostaining with 6E10 shows APPswe/ind levels are dramatically reduced in 6-mo-old mice treated for 1 wk with dox (upper panel). A separate blot was immunostained for APP C-terminal fragments with CT15 antibody to show that the precursors to Aβ cleavage are decreased in parallel with the full-length protein (middle panel). Costaining for superoxide dismutase 1 was used as an internal control for loading (lower panel, taken from bottom half of 6E10 blot).

To mimic therapeutic intervention with inhibitors of Aβ production, we raised a group of 25 double transgenic mice (CaMKIIα-tTA × APP line 107) on normal food until 6 mo of age, when we knew amyloid formation was already well underway in the brain. At 6 mo, half of the animals were switched from normal chow to food containing dox at 200 mg/kg until they were sacrificed at 9 or 12 mo of age. The remaining control animals were kept on standard chow (untreated). In all, four cohorts were created: 6 mo untreated (*n =* 7), 9 mo untreated (*n =* 5), 6 mo + 3 mo treated (*n =* 8), and 6 mo + 6 mo treated (*n =* 5). Full suppression (>95%) of transgenic APPswe/ind levels in the dox-treated animals was confirmed by immunoblot (Figure 3). To ensure that the transgene could be suppressed as rapidly in 6-mo-old mice with fulminant pathology as it can in young, predeposit animals, we treated an additional set of 6-mo-old animals with dox for 1 wk prior to harvest. Importantly, both APPswe/ind and APP–C-terminal fragment levels were fully suppressed after only 1 wk of treatment, indicating that the in vivo half-life of APPswe/ind and its processed C-terminal fragments are relatively short (Figure 3D).

**Figure 4:**
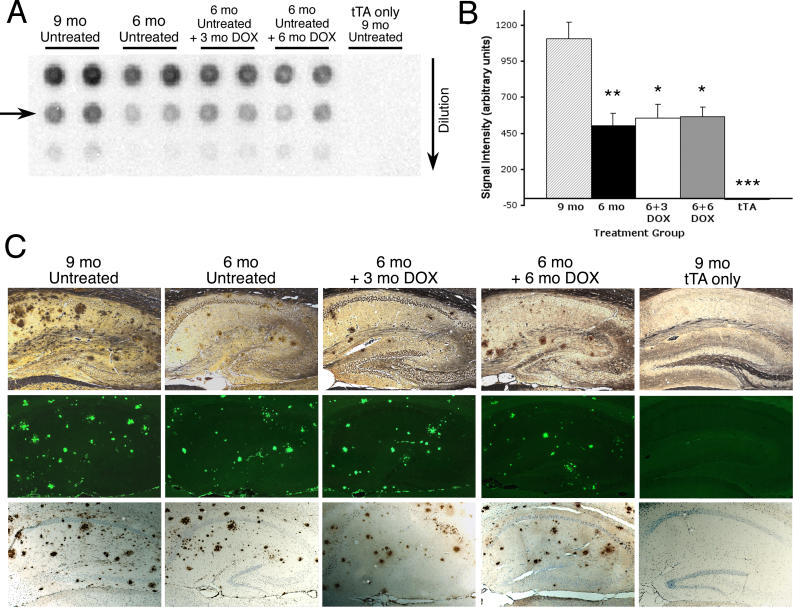
Suppression of Transgenic APP Arrests Progression of Amyloid Pathology (A) Aggregated Aβ was quantified in cortical tissue from dox-treated and control tTA/APP mice (line 107) using a filter trap assay. Serial dilutions of protein homogenate were passed through a cellulose acetate filter; protein aggregates larger than the pore size were trapped and immunostained for Aβ. (B) Quantitation of signal intensity in the linear range of each filter trap dilution series (arrow in [A]) was used to compare aggregate load across treatment groups. Aggregated Aβ increased significantly between 6 and 9 mo of age in untreated mice (significant effect of group ANOVA *F*_3,18_ = 7.85, *p <* 0.002). This progression of pathology was completely prevented by transgene suppression. The amount of aggregated Aβ was identical in untreated mice at 6 mo of age to that in 9- or 12-mo-old animals treated with dox (*p >* 0.9, Tukey post-hoc test). Single transgenic tTA samples were included as negative controls and showed no signal above background. *, *p <* 0.01; **, *p <* 0.005 versus 9-mo-old untreated mice, Tukey post-hoc test; ***, *p <* 0.001 versus 9-mo-old untreated mice, Student's *t*-test. (C) Amyloid pathology in the hippocampus of representative mice from each treatment group: Hirano silver stain (top row), thioflavin-S (middle row), and Aβ immunohistochemistry (bottom row). Amyloid burden increases dramatically between 6 and 9 mo of age in untreated animals, but remains stable in transgene-suppressed mice over the same period (6 mo + 3 mo dox and 6 mo + 6 mo dox). Single transgenic animals (tTA only shown here) show no sign of amyloid pathology at any age tested.

Tissue sections from each animal in the four treatment groups were stained for amyloid pathology by Hirano silver, Campbell-Switzer silver, thioflavin-S, and Aβ immunohistochemistry. As expected, the 6 mo untreated cohort displayed moderate amyloid pathology, and the 9 mo untreated cohort progressed to a severe amyloid burden. In contrast, the extent of amyloid pathology in mice from the 6 mo + 3 mo treated or 6 mo + 6 mo treated cohorts closely resembled that of the 6 mo untreated cohort, despite the significant age difference between the treated and untreated groups (Figures 4 and Figure S3). Well-formed plaques remained in the treated animals after 6 mo of transgene suppression, even though as much time was given to clear the lesions as they had taken to form. Moreover, both types of amyloid, diffuse and fibrillar, remained intact throughout treatment. Using the Campbell-Switzer silver stain to distinguish different forms of amyloid, we found diffuse plaques were as persistent as cored deposits (Figure S4). It was nevertheless clear that dox-induced suppression of transgenic APP had completely halted the progression of pathology.

To confirm that the arrest of plaques without any sign of clearance was not unique to the line 107 mice, we repeated the dox-suppression experiment in a second line of tet-off APP mice (CaMKIIα-tTA × tet-APPswe/ind line 18; *n =* 22). Again, long-term dox treatment was begun at 6 mo of age, and mice were harvested after 3 mo of transgene suppression (6 mo untreated, *n =* 8; 9 mo untreated, *n =* 6; 6 mo + 3 mo treated, *n =* 8). Immunoblotting for APP confirmed full transgene suppression in the treated animals (Figure S5). As in the line 107 mice described above, amyloid burden worsened substantially in the untreated mice between 6 and 9 mo of age. Suppression of transgene expression abruptly arrested progression of pathology (Figure S6), but again without any sign of reduction. Both silver- and thioflavin-S-positive plaques could still be found in each of the dox-treated animals.

We biochemically measured the amount of aggregated Aβ in the brains of our mice before and after transgene suppression using filter trap analysis of cortical tissue from each animal. In this assay, serial dilutions of protein homogenate are passed through a cellulose acetate filter; particles larger than the pore size of the filter become trapped in the membrane and are revealed by immunoblotting [22]. Consistent with our visual analysis of the histological sections, line 107 tTA/APP mice treated with dox for 3 or 6 mo had the same amount of aggregated Aβ as when they started treatment at 6 mo of age (Figure 4A and 4B). In contrast, untreated 9-mo-old mice had almost twice as much aggregated Aβ as either of the treated groups. Filter trap analysis of line 18 tTA/APP mice yielded similar results: the increase in aggregated Aβ observed in untreated animals between 6 and 9 mo of age was completely arrested by transgene suppression (Figure S5C and S5D).

**Figure 5:**
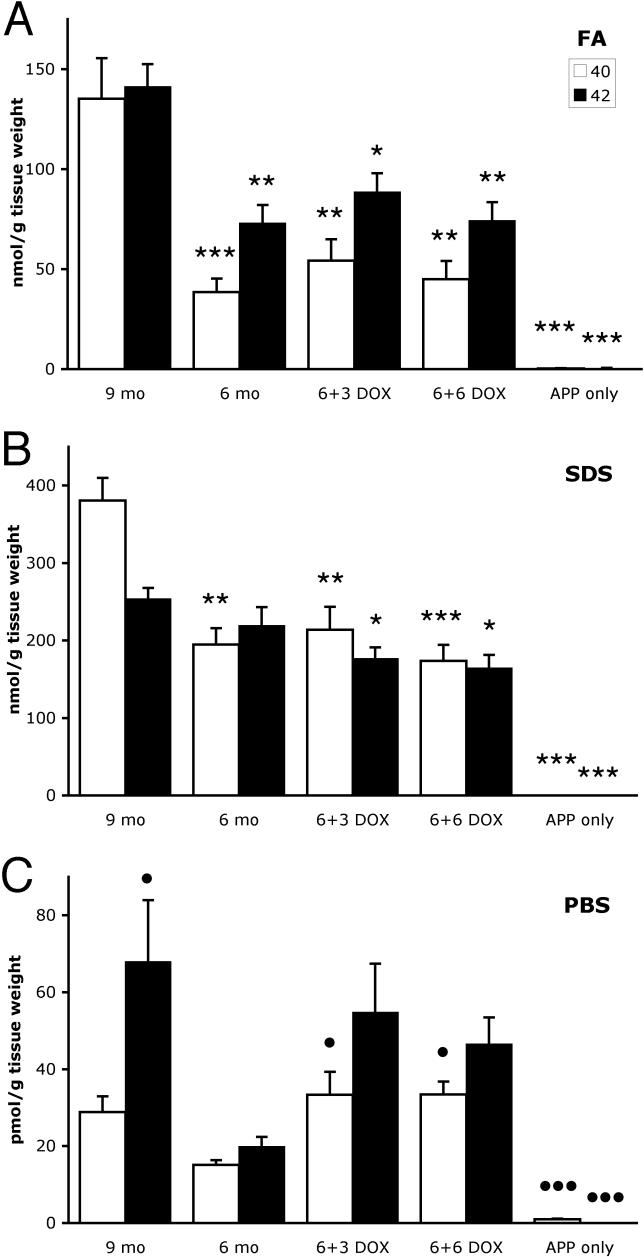
Aβ ELISA Confirms Arrest of Progression without Clearance of Peptide in Mice with Preexisting Aggregates Aβ levels in untreated 6- and 9-mo-old tTA/APP line 107 mice (shown in Figure 4) were compared to those in 9- and 12-mo-old animals treated with dox from the age of 6 mo. Single transgenic APP samples were included as negative controls. Cortical homogenates were fractionated by sequential multi-step extraction with PBS, 2% SDS, and 70% FA followed by human-specific Aβ ELISA to measure transgene-derived peptide in each fraction. Aβ40 is shown in white, Aβ42 in black. (A and B) Most Aβ in the brains of plaque-bearing mice is extracted into the FA and SDS fractions. Consistent with amyloid burden (Figures 4 and Figure S3), SDS- and FA-extracted Aβ levels in untreated 9-mo-old mice were significantly higher than in untreated 6-mo-old mice (Tukey post-hoc test applied to significant effect of group ANOVA for SDS and FA fractions *F*_3,18_ = 4.72–12.92, *p <* 0.02). In contrast, 3 or 6 mo of transgene suppression held Aβ at levels equivalent to those harbored when treatment was started (*p >* 0.2 compared to 6 mo untreated mice, Tukey post-hoc test). *, *p <* 0.05; **, *p <* 0.005; ***, *p <* 0.001 versus 9-mo-old untreated mice, Tukey post-hoc test. Significance for APP versus 9-mo-old untreated mice is based on Student's *t*-test. (C) The PBS fraction represents less than 0.1% of total Aβ (note the change in *y*-axis from [A] and [B]), but only here do Aβ levels in the dox-treated mice differ from those in younger untreated mice. Although both peptides appear elevated in the treated groups compared to the untreated 6-mo-old mice, only Aβ40 reaches statistical significance (*p <* 0.05, Tukey post-hoc test applied to significant effect of group ANOVA for Aβ40 *F*_3,18_ = 4.60, *p <* 0.02). A similar trend was seen for Aβ42, where ANOVA yielded a significant effect of group for PBS-soluble Aβ42 (*F*_3,18_ = 3.75, *p <* 0.03), however this was due only to differences between the untreated 6- and 9-mo-old groups. •, *p <* 0.05 versus 6-mo-old untreated mice, Tukey post-hoc test; •••, *p <* 0.001 versus 6-mo-old untreated mice, Student's *t*-test.

We next used ELISA to measure total Aβ in the brains of each group to determine whether any change in the amount or solubility of peptide occurred while APPswe/ind expression was suppressed. Cortical homogenates were sequentially extracted to separate peptide into PBS-, SDS-, and FA-soluble fractions, then transgene-derived Aβ40 and Aβ42 were measured by human-specific ELISA [23]. In all animals harboring amyloid deposits, we found that the vast majority of Aβ (>99%) was extracted into the SDS and FA fractions (Figure 5A and 5B). Consistent with the filter trap results presented above, there were no significant differences in SDS- or FA-soluble Aβ between the 6 mo untreated cohort and either the 6 mo + 3 mo treated or 6 mo + 6 mo treated cohorts. However, brains of both 6 mo + 3 mo and 6 mo + 6 mo treated cohorts contained roughly twice as much PBS-soluble Aβ40 as untreated 6-mo-old mice (Figure 5C). Levels of Aβ42 showed a similar trend, but did not reach statistical significance. In fact, levels of PBS-soluble Aβ40 and Aβ42 in the 6 mo + 3 mo and 6 mo + 6 mo treated cohorts were most similar to that of the 9 mo untreated cohort, suggesting that age, as opposed to synthetic rate (which would be negligible in the treated animals), may determine the fraction of PBS-soluble Aβ in these animals.

**Figure 6:**
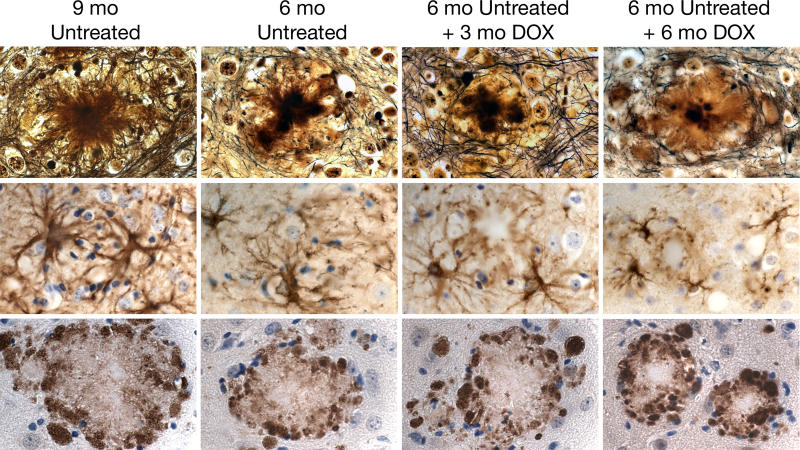
Neuritic and Glial Pathology Are Unchanged following Transgene Suppression Dystrophic neurites and activated astrocytes surround most compact plaques in tet-off APP mice (line 107). Dark-stained, ubiquitin-filled neurites and reactive astrocytes form a halo around cored, fibrillar deposits by 6 mo of age that worsens with time in untreated mice. Both plaque-associated pathologies are arrested, although not reversed, by transgene suppression. Hirano silver stain (top row); GFAP immunohistochemistry (middle row); ubiquitin immunohistochemistry (bottom row).

We also assessed neuritic and glial pathology surrounding the plaques to determine whether there were any changes in nearby tissue following long-term transgene suppression. Both Hirano silver stain and ubiquitin immunostaining showed neuritic pathology in all treatment groups (Figure 6). Similarly, activated astrocytes immunostained for GFAP were found near plaques in all animals (Figure 6). Neuritic and glial pathology were more severe in the older untreated mice. In contrast, transgene suppression prevented the growth of individual deposits apparent in untreated mice, and limited the surrounding pathology to what was already present when treatment began.

An obvious question we sought to address was whether the deposition of Aβ diminished cognitive ability in untreated mice, and what might happen to cognition when the process was interrupted. Unfortunately, efforts to characterize cognitive behavior were compromised by severe hyperactivity in untreated double transgenic mice. The tTA/APP animals were often seen running in circles around the perimeter of their cages, and a similar swimming pattern was noted when the mice were tested in the Morris water maze. In the radial water maze, repetitive swim patterns were noted with no evidence of choice-motivated actions. Other studies have dealt with similar problems by excluding animals that do not show adequate attention to the task, retaining only those mice that meet certain performance criteria [30]. In our case, the penetrance of hyperactivity was close to 100%, leaving us with no testable animals. This phenotype has not affected previous lines of APPswe mice we have produced, such as lines E1–2 or C3–3, that express lower levels of transgenic protein. Indeed, in past studies where hyperactivity was not a factor, we established a clear relationship between amyloid load and cognitive ability [31]. However, in the current study, we feel that although the poor performance of the tTA/APP mice in the maze tests could technically be scored as cognitive impairment, the animals' severe hyperactivity made interpretation of the cognitive tasks impossible.

**Figure 7:**
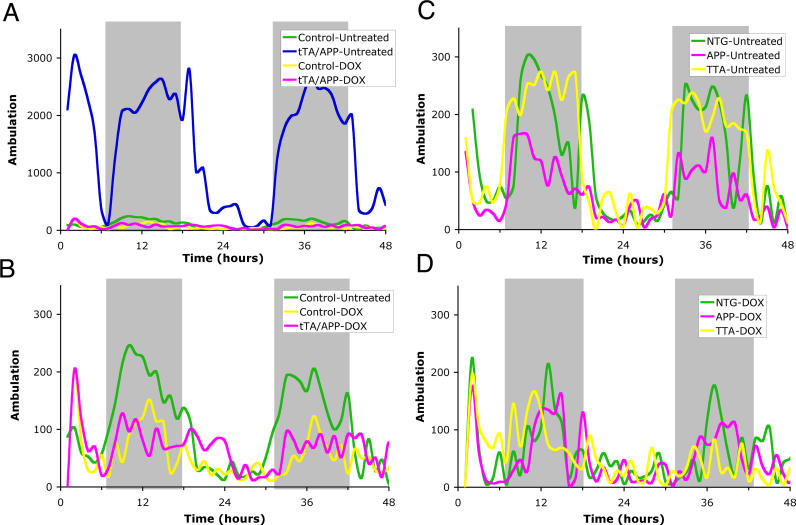
Transgene Suppression Attenuates Hyperactivity in tTA/APP Mice (A) A 48-h measure of ambulation records extreme hyperactivity in untreated double transgenic mice compared to single transgenic and nontransgenic controls (line 107). This phenotype is completely eliminated by rearing the double transgenic mice on dox. (B) The same data shown in (A) are replotted to magnify data from untreated control and dox-treated groups. (C and D) Activity levels in the combined control groups of (A) and (B) are here separated by genotype. None of the single transgenic or nontransgenic control groups display the hyperactivity present in untreated tTA/APP animals. Again, note the *y*-axes have been enlarged for detail compared to (A).

In order to understand the nature and extent of hyperactivity in the tTA/APP mice, we quantified daily activity levels in double transgenic animals along with their single transgenic and nontransgenic siblings using four-beam frames designed to monitor ambulation within an enclosed cage. As shown in Figure 7, the double transgenic mice were up to 10-fold more active during the dark phase of the day–night cycle than any of the control groups. Activity levels appeared to follow a relatively normal diurnal cycle, decreasing substantially during the daylight hours. However, even during the light phase, the tTA/APP mice remained many-fold more active than normal controls. This behavior was partially, but not consistently, reversed by 1 mo of transgene suppression beginning at 4–5 mo of age (data not shown). In contrast, hyperactivity was completely abolished by rearing tTA/APP mice on dox. Animals born and raised on dox showed activity levels similar to the untreated controls (Figure 7C). Intriguingly, all of the dox-reared animals, both transgenic and wild-type, showed altered circadian rhythms with far less distinction between their day- and nighttime activity levels.

## Discussion

We present a new mouse model for AD that was designed to test the consequences of inhibiting Aβ production after the onset of amyloid pathology. New lines of transgenic mice were developed for this study that express high levels of APPswe/ind under the control of a tetracycline-responsive promoter. We demonstrate that treatment with dox suppresses steady-state levels of both APPswe/ind and its C-terminal fragments, indicating that the mutant proteins have a relatively short half-life in vivo. Transgenic expression of APPswe/ind and consequent overproduction of Aβ42 cause early-onset amyloid deposition in untreated mice, in which deposits appear as early as 2 mo of age. Amyloid burden worsens significantly with age, and by 9 mo, the hippocampus and cortex of untreated mice are largely filled with aggregated peptide. We find that suppression of transgenic APP by more than 95% abruptly halts the progression of amyloid pathology. Importantly, this outcome occurs in animals already harboring considerable amyloid pathology, a situation similar to what might be expected in patients to be treated with secretase inhibitors. Somewhat unexpectedly, we observe no appreciable clearance of deposited amyloid even following periods of transgene suppression equal to the time taken for plaques to form. This latter finding indicates that compared to other disease-associated protein aggregates such as mutant huntingtin, which clear in less than 3 mo [32], the disaggregation of extracellular amyloid is relatively slow.

Notably, pharmaceutical γ-secretase inhibitors published to date show less strict regulation of Aβ production following chronic administration than we have attained here. Two independent compounds tested in Tg2576 [33] and TgCRND8 [34] transgenic mice show no more than 85% suppression of Aβ40 levels, and where measured, even less suppression of Aβ42 (approximately 60%) [35,36]. Thus, even after accounting for the much higher APP expression levels in our mice than in the Tg2576 and TgCRND8 lines, we have achieved better absolute suppression of Aβ production with the tet-off system than is currently possible with published γ-secretase inhibitors. Since even the most advanced future pharmaceutical agents are unlikely to attain more complete control of Aβ production than achieved here, this system provides a salient test of therapeutic intervention with Aβ-lowering compounds.

Although the progression of amyloid deposition was sharply arrested by this approach, we found that a substantial amyloid burden remained even after long periods of transgene suppression. We examined a small number of animals after 12 mo of dox treatment (beginning at 6 mo of age), and found that amyloid deposits were still relatively abundant. Longer-term treatments are now in progress. At the latest treatment interval analyzed by ELISA, animals administered dox for 6 mo showed elevated levels of PBS-soluble Aβ (see Figure 5) that could be interpreted as an indication that the plaques are slowly releasing peptide (or oligomeric Aβ) into the soluble pool and might eventually dissolve. Whether inhibiting Aβ production longer than 6 (or 12) mo may ultimately result in clearance of amyloid is under investigation; unfortunately, the life span of the model eventually limits this experiment. Therapeutics used in humans will have considerably more time to act than is possible within the life span of rodent models. Long-term treatments would certainly be possible and could be a key to effective therapy. Overall, however, we interpret our findings as evidence that AD therapies that significantly lower the production of Aβ (by either inhibiting secretase activity or inhibiting APP expression) may not quickly reverse preexisting pathology, but should effectively halt further deposition of amyloid.

In interpreting our study, it should be remembered that the earliest plaques to appear in these mice, like other APP transgenics harboring the Swedish mutation [27], are predominantly fibrillar deposits, which may be less tractable than the diffuse aggregates thought to come first in the course of the human disease. However, our data suggest that once diffuse deposits are formed in these mice, they are no more easily cleared in our system than cored plaques (see Figure S4). An additional consideration we recognize is that a small amount of transgene expression continues in the presence of dox and that endogenous mouse Aβ continues to be produced. It is possible that the combined low levels of endogenous mouse Aβ and nonsuppressed human peptide are sufficient to maintain existing deposits. However, these low levels of peptide are not sufficient to induce new amyloid formation, as CaMKIIα-tTA × tetAPPswe/ind mice raised on dox for up to a year do not develop amyloid lesions (data not shown). It is also clear that in this genetic system, we have raised the production of Aβ to levels not found in humans to accelerate pathology into an experimentally feasible time frame. This system allowed us to create an approximately 20-fold differential between APP/Aβ synthetic rates before and after treatment, yet the in vivo equilibrium between aggregated and disaggregated states of Aβ still favored the maintenance of amyloid deposits. In our opinion, it seems unlikely that amyloid deposits in human brain would be inherently any less stable than those formed in mouse brain. However, the human brain may harbor clearance mechanisms not shared by mice that would allow more efficient removal of preexisting amyloid.

One potential mechanism by which amyloid may be more efficiently removed in the human disease than in the mouse models is through microglial phagocytosis. Resident microglia in transgenic mouse models localize to tissue surrounding plaques but show little evidence of amyloid engulfment [37–40]. In contrast, microglia surrounding amyloid plaques in human brain show a much higher state of activation with greater expression of complement receptor [40]. Thus, the role of microglia in amyloid metabolism is minor in transgenic models compared to the human condition. Somewhat paradoxically, several studies further demonstrate that treatment with anti-inflammatory drugs to reduce microglial activation actually lowers amyloid load in APP transgenic mice, suggesting a role for mouse microglia in the formation and maintenance of amyloid aggregates [41–43]. However, this outcome may be alternatively explained by direct effects of many anti-inflammatory drugs on γ-secretase cleavage [44–47]. Nonetheless, the role of microglia in both the human condition and the mouse models is poorly understood, and differences in microglial reactivity between the two could lead to significantly faster amyloid clearance in the brains of patients with AD than we observe in the tet-off APP mice.

Given the relatively minor role played by microglia in other mouse models of amyloidosis, we think it unlikely that these cells have influenced the rate of amyloid clearance in the tet-off APP mice. Even so, we considered the possibility that chronic dox treatment may have altered the activation state of microglia in our treated mice. Dox is structurally similar to minocycline, a reported anti-inflammatory drug and inhibitor of microglial activation [48]. However, if dox does have anti-inflammatory activity, then, based on previous studies with other anti-inflammatories, we would have expected to find less amyloid in the dox-treated animals. Clearly, that was not the case. While it is possible that dox acts in some other way to slow amyloid clearance, data from multiple studies demonstrate that microglial responses are normally weak in the mouse AD models [37–40], and thus it is doubtful that dox-mediated microglial inhibition affected the outcome of our study.

The persistence and stability of amyloid deposits in our system is unexpected given the speed with which Aβ aggregates are cleared in other mouse models of therapeutic intervention. Anti-Aβ antibodies injected directly into the brain have been shown to eliminate amyloid deposits in as little as 1 wk after treatment [49–51]. Peripheral antibody injection decreases amyloid load more broadly, and although it does not appear to act as quickly as local injection, can significantly reduce amyloid load within 2 mo of initial treatment [52,53]. More recently, an alternative approach has shown that lentiviral transfer of neprilysin can also reduce the number of aggregates in the area of the injection site [54]. Careful study of the mechanism behind several of the antibody-mediated therapies has suggested that activated microglia play an important role in the removal of fibrillar plaques after immunization [50,52,55]. However, it has been noted that deletion of the Fc receptor (the primary receptor for microglial opsinization of antibody–antigen complexes) in APP transgenic mouse models has no impact on the effectiveness of antibody-mediated therapy [56,57]. It is, nevertheless, possible that lack of microglia activation is the major difference between the slow clearance described here, where no perturbation of the immune system is expected, and the rapid clearance described in studies involving antibody or viral injection. In isolation, mild activation of microglia by injection damage or opsinization may not be adequate to induce substantial phagocytosis, but when combined with an Aβ-lowering agent, such as neprilysin or Aβ-targeted antibodies, the two may work in concert to clear peptide deposits. Consistent with this hypothesis, strong activation of microglia through transgenic expression of TGFβ [58] or central injection of lipopolysaccharide [59,60] can by itself substantially reduce plaque burden in APP transgenic mice. But in the case of acute antibody- and/or injury-mediated activation, once the inflammation has passed, and the antibody and bound peptide have been cleared and degraded, the remaining Aβ quickly reaggregates and amyloid pathology is reestablished [49]. This finding reinforces the notion that without continued stimulation, microglia in mouse models do not maintain the same level of sustained activation that may occur in humans.

SantaCruz et al. recently published a study of mice that express P301L human tau via a similar vector system [61]. As in our tet-off APP mice, SantaCruz et al. found that tau neurofibrillary tangles, like amyloid plaques, are not cleared efficiently following transgene suppression. The lack of clearance in both models of AD pathology comes as a stark contrast to the rapid removal of protein aggregates found in similar tet-off mouse models of Huntington [32] and prion disease [62]. In these cases, disrupting the input of new monomer to the system via dox-mediated transgene suppression led to relatively rapid clearance of protein aggregates. By contrast, our study and that of SantaCruz et al. suggest that protein aggregates in AD may be more tenacious than in other neurodegenerative disorders. Perhaps once aggregated, Aβ and tau are either inherently more stable than other protein aggregates or more resistant to intra- and extracellular clearance mechanisms.

One question we were not able to address in this study is whether abrogating synthesis of new Aβ halts the progression of cognitive decline. Studies from the tet-off tau mice suggest that protein clearance may in fact not be required for cognitive improvement following transgene suppression [61]. At present, because of unexpected noncognitive behavioral abnormalities, it is not clear whether the tet-off APP mice can be used to address the same question in the context of amyloid pathology. Both lines of tTA/APP mice we studied here display extreme hyperactivity visible as cage circling and quantified by activity monitoring (see Figure 7). Many of the double transgenic mice showed similar circular patterns of swimming near the edge of the tank when tested in the Morris water maze. Expression of this phenotype makes standard tests of learning and memory uninterpretable. Hyperactivity nonspecifically inhibits choice-driven changes in movement, the key element behind all cognitive behavioral paradigms. We are currently working to determine whether hyperactivity correlates with expression of the APPswe/ind holoprotein or its proteolytic derivatives. Preliminary studies suggest that hyperactivity does not appear quickly when dox-reared mice are shifted to nonmedicated diets (J. L. J., unpublished data). These data may indicate that the neuroactive culprit is not immediately present after transgenic APP synthesis is initiated, but requires additional time to develop. Alternatively, hyperactivity may be caused by neuronal alterations due to transgene expression during early postnatal development. Further experiments are needed to distinguish between these possibilities.

In summary, we demonstrate that abrogating Aβ production halts the progression of pathologic changes in a transgenic mouse model of Alzheimer-type amyloidosis. However, despite dramatic reductions in Aβ synthesis, neuritic plaques are stable structures in vivo that do not quickly disaggregate. It is possible that a combination of therapies to limit Aβ production, increase Aβ degradation, and enhance phagocytosis of deposited amyloid may be required to reverse damage associated with AD. However, if started early enough in the course of disease, secretase inhibitors alone could provide substantial benefit in slowing pathogenic processes linked to amyloid deposition. Even at later stages in the disease, the presence of substantial microglial activation in human AD [40] suggests that simply slowing the formation of new amyloid deposits may allow ongoing phagocytosis to diminish preexisting lesions. However, the development of safe and effective secretase inhibitors will ultimately be required to determine whether the human brain has the capacity to repair amyloid-associated damage of AD once the progression of pathology is arrested.

## Supporting Information

Figure S1 Transgenic APP Expression and Suppression by Dox in the Four New Tet-Off APP Lines Western blotting with human-specific antibody 6E10 reveals transgene-derived full-length APP in cortical homogenates from untreated animals (left lanes of each panel). The new lines produce exceptionally high levels of transgene expression; an equal amount of brain homogenate from a standard transgenic APP line is shown for comparison (extreme left lane, Standard Tg line C3–3; [15,63]). After 1 mo of dox treatment, transgenic protein in all four new tet-off APP lines is reduced to residual levels (+ dox; right lanes of each panel).
(474 KB TIF).

Figure S2 Transgenic APP mRNA Is Brain-Specific A slot blot of mRNA harvested from various tissues in three of the four new tet-off APP lines and a nontransgenic control was used to examine transgenic mRNA expression. Hybridization is seen only in the brain; no signal above background is seen in any other tissue.
(781 KB PSD).

Figure S3 Amyloid Pathology in the Cortex Reiterates That in the Hippocampus Amyloid histology was performed on sections from line 107 double transgenic mice by Hirano silver stain (top row), thioflavin-S (middle row), and Aβ immunohistochemistry (bottom row) to examine the persistence of pathology following transgene suppression. As in the hippocampus (see Figure 4 and text), the progression of amyloid pathology in the cortex worsens substantially between 6 and 9 mo of age in untreated mice. This progression is completely prevented by suppression of the transgene with dox. For comparison, normal neurohistology is shown in an age-matched single transgenic (tTA only) animal. No amyloid pathology has been detected in either APP or tTA single transgenic animals up to 15 mo of age.
(4.8 MB PSD).

Figure S4 Diffuse Deposits Do Not Disperse During Aβ Suppression Campbell–Switzer silver stain was used to differentiate cored (brown) from diffuse (black) deposits in line 107 tTA/APP mice. This stain demonstrates that both types of deposit persist throughout long periods of transgene suppression. The lower panels, showing low-power images (10×) of frontal cortex from each condition, reveal little change in the extent of diffuse amyloid following up to 6 mo of Aβ suppression. High-power images (40×) in the upper panels show that the diffuse halo surrounding individual cored deposits remains relatively unchanged in treated mice. Untreated tTA single transgenic animals are shown as a negative control. Protocol for the Campbell-Switzer Alzheimer's Method was kindly shared by Robert Switzer, III (NeuroScience Associates, Knoxville, Tennessee, United States), and can be downloaded at http://www.nsalabs.com/Documents/publications/campbell-switzer_protocol.htm [64,65].
(923 KB JPG).

Figure S5 Chronic Transgene Suppression and Arrest of Aβ Aggregate Formation in an Independent Line of Tet-Off APP Mice (CaMKIIα-tTA × tet-APPswe/ind Line 18) (A) The experiment presented in the text for line 107 tet-off APP was repeated with a second tet-off APP line (line 18) to control for integration site artifacts. Cortical homogenates from untreated control and dox-treated double transgenic mice were immunoblotted for full-length APP with the human-specific antibody 6E10 to confirm transgene suppression at the time of harvest. Immunostaining for endogenous superoxide dismutase (SOD1) was included as a loading control.
(B) Quantitation of signal intensity from the Western blot in (A) shows transgenic APP levels in line 18 are suppressed by more than 98% following 3 mo of dox treatment (significant effect of group ANOVA *F*_2,8_ = 1559.7, *p <* 0.001). This level of suppression was equal to or better than that attained in line 107 (see Figure 3B).
(C) Serial dilution filter trap assay was used to quantify aggregated Aβ in cortical homogenates.
(D) Quantitation of signal intensity in the linear range of the dilution series shown in (C). Consistent with the amyloid histology shown in Figure S5, aggregate formation was significantly increased between 6 and 9 mo of age in untreated mice (significant effect of group ANOVA *F*_2,18_ = 12.14, *p <* 0.001). Aggregate formation was completely arrested by transgene suppression, and is identical in 9-mo-old mice treated with dox for 3 mo as in untreated animals harvested when treatment began (*p >* 0.5, Tukey post-hoc test). *, *p <* 0.05; **, *p <* 0.005; ***, *p <* 0.001 versus 9-mo-old untreated mice, Tukey post-hoc test.
(962 KB TIF).

Figure S6 Arrest of Amyloid Progression by Chronic Transgene Suppression in Line 18 Tet-Off APP Mice Amyloid histology in cortical (first and third rows) and hippocampal (second and fourth rows) sections from untreated tTA/APP mice shows a dramatic progression of pathology between 6 and 9 mo of age. Suppression of transgenic APP expression arrests this progression, although without any sign of plaque clearance (6 mo + 3 mo dox). Hirano silver stain (top panels); thioflavin-S (bottom panels).
(5.8 MB PSD).

**Patient Summary**

**Background**

Patients with Alzheimer disease (AD) have elevated levels of a small protein called amyloid-β peptide that sticks together to form what are known as amyloid plaques in their brains. This peptide is normally made at low levels in healthy individuals, and is made when a larger protein called amyloid precursor protein (APP) is cut down in size. New treatments are now being developed that will decrease the amount of Aβ produced from APP. However, it is not clear whether lowering the production of Aβ will allow the brain to heal itself by clearing the amyloid plaques. The answer to this question may be important for deciding when Aβ-lowering drugs should be started, and may also determine how effective they are in reversing the mental symptoms of AD.

**What Did the Researchers Do and Find?**

Because new drugs designed to lower Aβ levels are still in development, they are not available for testing in animal models of the disease. Instead, basic questions about the effectiveness of this type of treatment must be answered using systems that mimic how the drugs work. To do this, the authors created mice that produce too much APP and that develop the same amyloid lesions as do human patients with AD. Unlike normal mice, these mice also carried a “switch” gene that allowed the researchers to turn off APP by feeding the mice special food. Turning off APP in these mice had the same effect as treating them with Aβ-lowering drugs, and so the researchers were able to ask what happened to the amyloid plaques after Aβ production was shut down. They showed that lowering Aβ production prevents the amyloid lesions from getting worse as the disease progresses. This means that treatment with Aβ-lowering drugs may be able to stop the disease from filling the brain with plaques. However, the researchers also found that the amyloid lesions that had formed before treatment was started remained intact throughout the experiment.

**What Do These Findings Mean?**

These results indicate that treatments designed to lower the production of Aβ may be an important part of future AD treatment, as this approach seems to prevents additional amyloid plaques from forming in the mouse brain. However, by itself, this strategy may not be able to rid the brain of plaques that have already formed in the brain before treatment is started. The findings suggest that early treatment may be important for this approach to succeed.

**Where Can I Get More Information Online?**

MedlinePlus has several Web pages of information on Alzheimer disease:

http://www.nlm.nih.gov/medlineplus/alzheimersdisease.html

The ADEAR Center of the US Government's National Institute on Aging also has information on Alzheimer disease:

http://www.alzheimers.org/

The Alzheimer's Association Web site contains information on both caregiving and research:

http://www.alz.org

## Abbreviations

Aβ: amyloid-β
AD: Alzheimer disease
APLP: amyloid precursor–like protein
APP: amyloid precursor protein
CaMKIIα: calcium-calmodulin kinase IIα
dox: doxycycline
FA: formic acid
GFAP: glial fibrillary acidic protein
mo/huAPP695: mouse APP with a humanized Aβ domain
PBS: phosphate-buffered saline
SDS: sodium dodecyl sulfate
swe/ind: Swedish/Indiana
tTA: tetracycline transactivator
